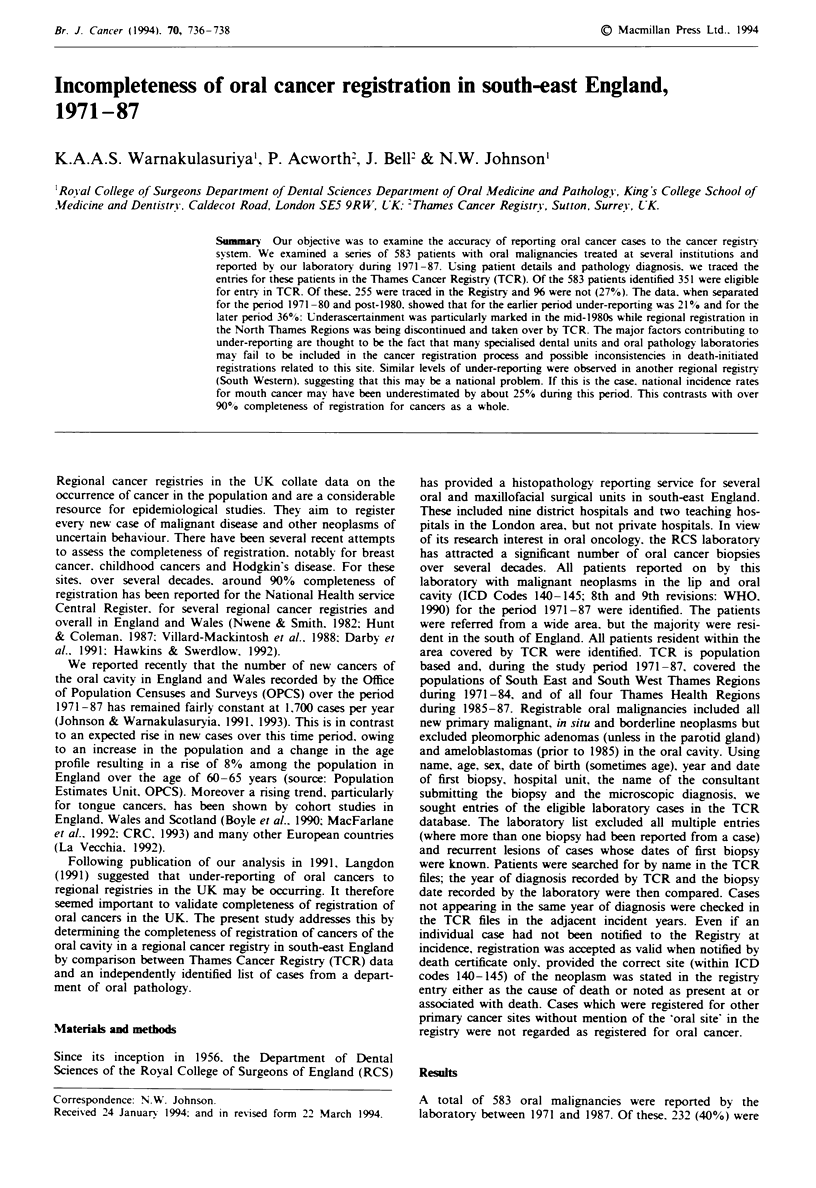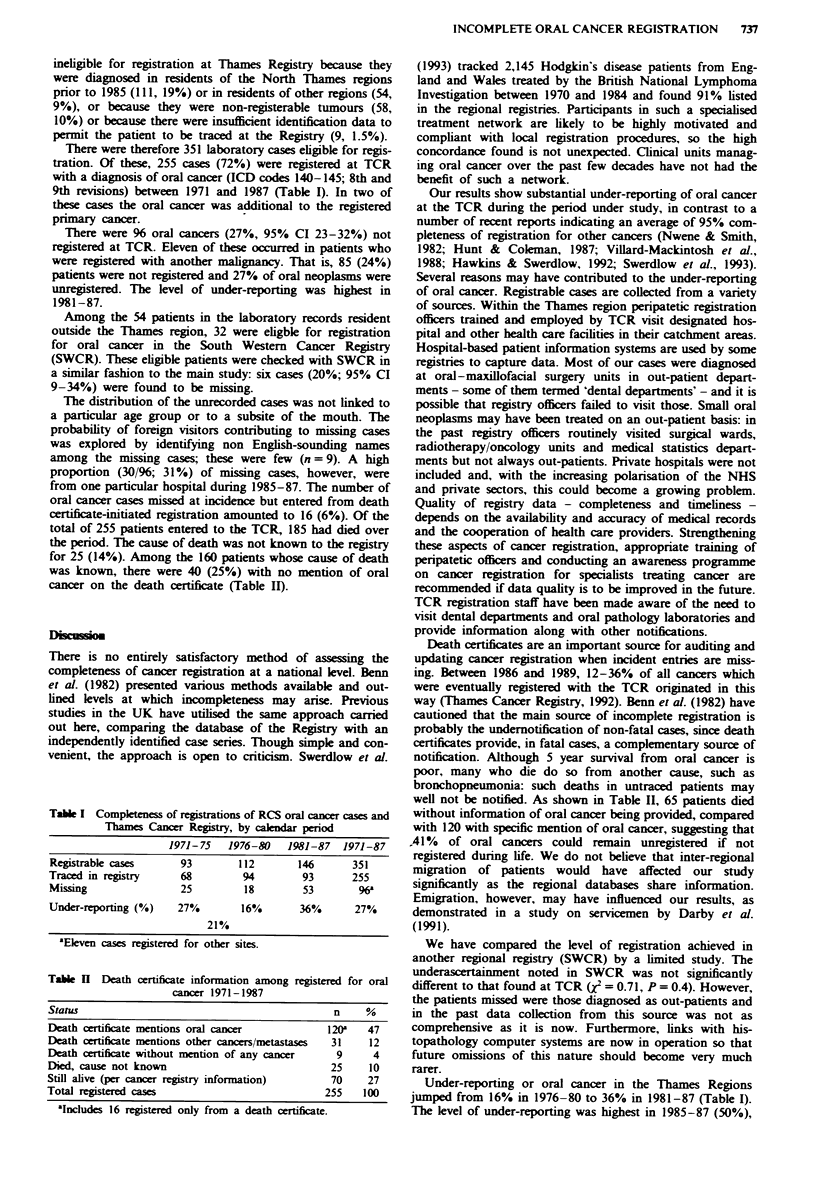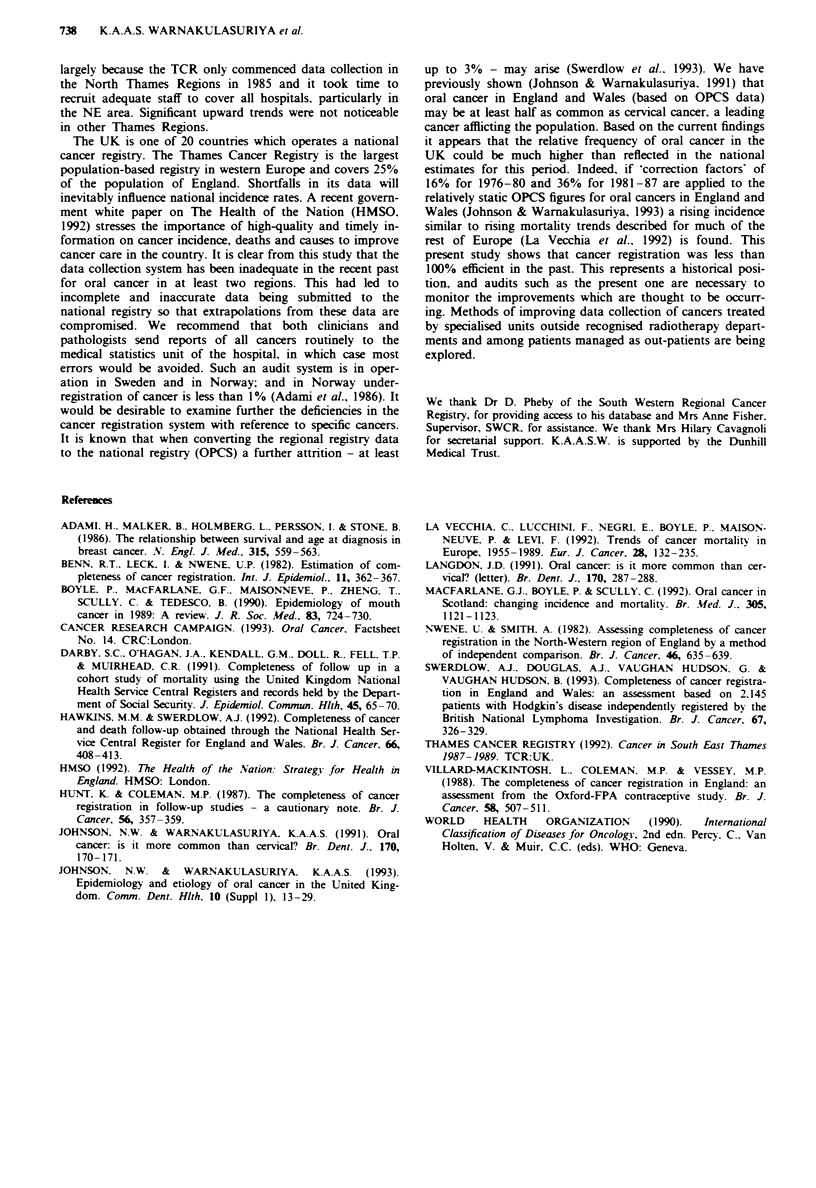# Incompleteness of oral cancer registration in south-east England, 1971-87.

**DOI:** 10.1038/bjc.1994.386

**Published:** 1994-10

**Authors:** K. A. Warnakulasuriya, P. Acworth, J. Bell, N. W. Johnson

**Affiliations:** Royal College of Surgeons Department of Dental Sciences, London, UK.

## Abstract

Our objective was to examine the accuracy of reporting oral cancer cases to the cancer registry system. We examined a series of 583 patients with oral malignancies treated at several institutions and reported by our laboratory during 1971-87. Using patient details and pathology diagnosis, we traced the entries for these patients in the Thames Cancer Registry (TCR). Of the 583 patients identified 351 were eligible for entry in TCR. Of these, 255 were traced in the Registry and 96 were not (27%). The data, when separated for the period 1971-80 and post-1980, showed that for the earlier period under-reporting was 21% and for the later period 36%: Underascertainment was particularly marked in the mid-1980s while regional registration in the North Thames Regions was being discontinued and taken over by TCR. The major factors contributing to under-reporting are thought to be the fact that many specialised dental units and oral pathology laboratories may fail to be included in the cancer registration process and possible inconsistencies in death-initiated registrations related to this site. Similar levels of under-reporting were observed in another regional registry (South Western), suggesting that this may be a national problem. If this is the case, national incidence rates for mouth cancer may have been underestimated by about 25% during this period. This contrasts with over 90% completeness of registration for cancers as a whole.


Br. J. Cancer (1994), 70, 736-738 © Macmillan Press Ltd., 1994

### Incompleteness of oral cancer registration in south-east England, 1971-87

K.A.A.S. Warnakulasuriya[1], P. Acworth[2], J. Bell[2] & N.W. Johnson[1]

[1]Royal College of Surgeons Department of Dental Sciences Department of Oral Medicine and Pathology, King's College School of Medicine and Dentistry, Caldecot Road, London SE5 9RW, UK; [2]Thames Cancer Registry, Sutton, Surrey, UK.

**Summary** Our objective was to examine the accuracy of reporting oral cancer cases to the cancer registry system. We examined a series of 583 patients with oral malignancies treated at several institutions and reported by our laboratory during 1971-87. Using patient details and pathology diagnosis, we traced the entries for these patients in the Thames Cancer Registry (TCR). Of the 583 patients identified 351 were eligible for entry in TCR. Of these, 255 were traced in the Registry and 96 were not (27%). The data, when separated for the period 1971-80 and post-1980, showed that for the earlier period under-reporting was 21% and for the later period 36%: Underascertainment was particularly marked in the mid-1980s while regional registration in the North Thames Regions was being discontinued and taken over by TCR. The major factors contributing to under-reporting are thought to be the fact that many specialised dental units and oral pathology laboratories may fail to be included in the cancer registration process and possible inconsistencies in death-initiated registrations related to this site. Similar levels of under-reporting were observed in another regional registry (South Western), suggesting that this may be a national problem. If this is the case, national incidence rates for mouth cancer may have been underestimated by about 25% during this period. This contrasts with over 90% completeness of registration for cancers as a whole.

Regional cancer registries in the UK collate data on the occurrence of cancer in the population and are a considerable resource for epidemiological studies. They aim to register every new case of malignant disease and other neoplasms of uncertain behaviour. There have been several recent attempts to assess the completeness of registration, notably for breast cancer, childhood cancers and Hodgkin's disease. For these sites, over several decades, around 90% completeness of registration has been reported for the National Health service Central Register, for several regional cancer registries and overall in England and Wales (Nwene & Smith, 1982; Hunt & Coleman, 1987; Villard-Mackintosh *et al.*, 1988; Darby *et al.*, 1991; Hawkins & Swerdlow, 1992).

We reported recently that the number of new cancers of the oral cavity in England and Wales recorded by the Office of Population Censuses and Surveys (OPCS) over the period 1971-87 has remained fairly constant at 1,700 cases per year (Johnson & Warnakulasuryia, 1991, 1993). This is in contrast to an expected rise in new cases over this time period, owing to an increase in the population and a change in the age profile resulting in a rise of 8% among the population in England over the age of 60-65 years (source: Population Estimates Unit, OPCS). Moreover a rising trend, particularly for tongue cancers, has been shown by cohort studies in England, Wales and Scotland (Boyle *et al.*, 1990; MacFarlane *et al.*, 1992; CRC, 1993) and many other European countries (La Vecchia, 1992).

Following publication of our analysis in 1991, Langdon (1991) suggested that under-reporting of oral cancers to regional registries in the UK may be occurring. It therefore seemed important to validate completeness of registration of oral cancers in the UK. The present study addresses this by determining the completeness of registration of cancers of the oral cavity in a regional cancer registry in south-east England by comparison between Thames Cancer Registry (TCR) data and an independently identified list of cases from a department of oral pathology.

### Materials and methods

Since its inception in 1956, the Department of Dental Sciences of the Royal College of Surgeons of England (RCS) has provided a histopathology reporting service for several oral and maxillofacial surgical units in south-east England. These included nine district hospitals and two teaching hospitals in the London area, but not private hospitals. In view of its research interest in oral oncology, the RCS laboratory has attracted a significant number of oral cancer biopsies over several decades. All patients reported on by this laboratory with malignant neoplasms in the lip and oral cavity (ICD Codes 140-145; 8th and 9th revisions: WHO, 1990) for the period 1971-87 were identified. The patients were referred from a wide area, but the majority were resident in the south of England. All patients resident within the area covered by TCR were identified. TCR is population based and, during the study period 1971-87, covered the populations of South East and South West Thames Regions during 1971-84, and of all four Thames Health Regions during 1985-87. Registrable oral malignancies included all new primary malignant, *in situ* and borderline neoplasms but excluded pleomorphic adenomas (unless in the parotid gland) and ameloblastomas (prior to 1985) in the oral cavity. Using name, age, sex, date of birth (sometimes age), year and date of first biopsy, hospital unit, the name of the consultant submitting the biopsy and the microscopic diagnosis, we sought entries of the eligible laboratory cases in the TCR database. The laboratory list excluded all multiple entries (where more than one biopsy had been reported from a case) and recurrent lesions of cases whose dates of first biopsy were known. Patients were searched for by name in the TCR files; the year of diagnosis recorded by TCR and the biopsy date recorded by the laboratory were then compared. Cases not appearing in the same year of diagnosis were checked in the TCR files in the adjacent incident years. Even if an individual case had not been notified to the Registry at incidence, registration was accepted as valid when notified by death certificate only, provided the correct site (within ICD codes 140-145) of the neoplasm was stated in the registry entry either as the cause of death or noted as present at or associated with death. Cases which were registered for other primary cancer sites without mention of the 'oral site' in the registry were not regarded as registered for oral cancer.

### Results

A total of 583 oral malignancies were reported by the laboratory between 1971 and 1987. Of these, 232 (40%) were

Correspondence: N.W. Johnson.

Received 24 January 1994; and in revised form 22 March 1994.

ineligible for registration at Thames Registry because they were diagnosed in residents of the North Thames regions prior to 1985 (111, 19%) or in residents of other regions (54, 9%), or because they were non-registerable tumours (58, 10%) or because there were insufficient identification data to permit the patient to be traced at the Registry (9, 1.5%).

There were therefore 351 laboratory cases eligible for registration. Of these, 255 cases (72%) were registered at TCR with a diagnosis of oral cancer (ICD codes 140–145; 8th and 9th revisions) between 1971 and 1987 (Table I). In two of these cases the oral cancer was additional to the registered primary cancer.

There were 96 oral cancers (27%, 95% CI 23–32%) not registered at TCR. Eleven of these occurred in patients who were registered with another malignancy. That is, 85 (24%) patients were not registered and 27% of oral neoplasms were unregistered. The level of under-reporting was highest in 1981–87.

Among the 54 patients in the laboratory records resident outside the Thames region, 32 were eligble for registration for oral cancer in the South Western Cancer Registry (SWCR). These eligible patients were checked with SWCR in a similar fashion to the main study: six cases (20%; 95% CI 9–34%) were found to be missing.

The distribution of the unrecorded cases was not linked to a particular age group or to a subsite of the mouth. The probability of foreign visitors contributing to missing cases was explored by identifying non English-sounding names among the missing cases; these were few ($n = 9$). A high proportion (30/96; 31%) of missing cases, however, were from one particular hospital during 1985–87. The number of oral cancer cases missed at incidence but entered from death certificate-initiated registration amounted to 16 (6%). Of the total of 255 patients entered to the TCR, 185 had died over the period. The cause of death was not known to the registry for 25 (14%). Among the 160 patients whose cause of death was known, there were 40 (25%) with no mention of oral cancer on the death certificate (Table II).

### Discussion

There is no entirely satisfactory method of assessing the completeness of cancer registration at a national level. Benn *et al.* (1982) presented various methods available and outlined levels at which incompleteness may arise. Previous studies in the UK have utilised the same approach carried out here, comparing the database of the Registry with an independently identified case series. Though simple and convenient, the approach is open to criticism. Swerdlow *et al.* (1993) tracked 2,145 Hodgkin's disease patients from England and Wales treated by the British National Lymphoma Investigation between 1970 and 1984 and found 91% listed in the regional registries. Participants in such a specialised treatment network are likely to be highly motivated and compliant with local registration procedures, so the high concordance found is not unexpected. Clinical units managing oral cancer over the past few decades have not had the benefit of such a network.

**Table I** Completeness of registrations of RCS oral cancer cases and Thames Cancer Registry, by calendar period

| | *1971–75* | *1976–80* | *1981–87* | *1971–87* |
|---|---|---|---|---|
| Registrable cases | 93 | 112 | 146 | 351 |
| Traced in registry | 68 | 94 | 93 | 255 |
| Missing | 25 | 18 | 53 | 96[a] |
| Under-reporting (%) | 27% | 16% | 36% | 27% |
| | 21% | | | |

[a]Eleven cases registered for other sites.

**Table II** Death certificate information among registered for oral cancer 1971–1987

| *Status* | n | % |
|---|---|---|
| Death certificate mentions oral cancer | 120[a] | 47 |
| Death certificate mentions other cancers/metastases | 31 | 12 |
| Death certificate without mention of any cancer | 9 | 4 |
| Died, cause not known | 25 | 10 |
| Still alive (per cancer registry information) | 70 | 27 |
| Total registered cases | 255 | 100 |

[a]Includes 16 registered only from a death certificate.

Our results show substantial under-reporting of oral cancer at the TCR during the period under study, in contrast to a number of recent reports indicating an average of 95% completeness of registration for other cancers (Nwene & Smith, 1982; Hunt & Coleman, 1987; Villard-Mackintosh *et al.*, 1988; Hawkins & Swerdlow, 1992; Swerdlow *et al.*, 1993). Several reasons may have contributed to the under-reporting of oral cancer. Registrable cases are collected from a variety of sources. Within the Thames region peripatetic registration officers trained and employed by TCR visit designated hospital and other health care facilities in their catchment areas. Hospital-based patient information systems are used by some registries to capture data. Most of our cases were diagnosed at oral–maxillofacial surgery units in out-patient departments – some of them termed 'dental departments' – and it is possible that registry officers failed to visit those. Small oral neoplasms may have been treated on an out-patient basis: in the past registry officers routinely visited surgical wards, radiotherapy/oncology units and medical statistics departments but not always out-patients. Private hospitals were not included and, with the increasing polarisation of the NHS and private sectors, this could become a growing problem. Quality of registry data – completeness and timeliness – depends on the availability and accuracy of medical records and the cooperation of health care providers. Strengthening these aspects of cancer registration, appropriate training of peripatetic officers and conducting an awareness programme on cancer registration for specialists treating cancer are recommended if data quality is to be improved in the future. TCR registration staff have been made aware of the need to visit dental departments and oral pathology laboratories and provide information along with other notifications.

Death certificates are an important source for auditing and updating cancer registration when incident entries are missing. Between 1986 and 1989, 12–36% of all cancers which were eventually registered with the TCR originated in this way (Thames Cancer Registry, 1992). Benn *et al.* (1982) have cautioned that the main source of incomplete registration is probably the undernotification of non-fatal cases, since death certificates provide, in fatal cases, a complementary source of notification. Although 5 year survival from oral cancer is poor, many who die do so from another cause, such as bronchopneumonia: such deaths in untraced patients may well not be notified. As shown in Table II, 65 patients died without information of oral cancer being provided, compared with 120 with specific mention of oral cancer, suggesting that 41% of oral cancers could remain unregistered if not registered during life. We do not believe that inter-regional migration of patients would have affected our study significantly as the regional databases share information. Emigration, however, may have influenced our results, as demonstrated in a study on servicemen by Darby *et al.* (1991).

We have compared the level of registration achieved in another regional registry (SWCR) by a limited study. The underascertainment noted in SWCR was not significantly different to that found at TCR ($\chi^2 = 0.71$, $P = 0.4$). However, the patients missed were those diagnosed as out-patients and in the past data collection from this source was not as comprehensive as it is now. Furthermore, links with histopathology computer systems are now in operation so that future omissions of this nature should become very much rarer.

Under-reporting or oral cancer in the Thames Regions jumped from 16% in 1976–80 to 36% in 1981–87 (Table I). The level of under-reporting was highest in 1985–87 (50%), largely because the TCR only commenced data collection in the North Thames Regions in 1985 and it took time to recruit adequate staff to cover all hospitals, particularly in the NE area. Significant upward trends were not noticeable in other Thames Regions.

The UK is one of 20 countries which operates a national cancer registry. The Thames Cancer Registry is the largest population-based registry in western Europe and covers 25% of the population of England. Shortfalls in its data will inevitably influence national incidence rates. A recent government white paper on The Health of the Nation (HMSO, 1992) stresses the importance of high-quality and timely information on cancer incidence, deaths and causes to improve cancer care in the country. It is clear from this study that the data collection system has been inadequate in the recent past for oral cancer in at least two regions. This had led to incomplete and inaccurate data being submitted to the national registry so that extrapolations from these data are compromised. We recommend that both clinicians and pathologists send reports of all cancers routinely to the medical statistics unit of the hospital, in which case most errors would be avoided. Such an audit system is in operation in Sweden and in Norway; and in Norway under-registration of cancer is less than 1% (Adami et al., 1986). It would be desirable to examine further the deficiencies in the cancer registration system with reference to specific cancers. It is known that when converting the regional registry data to the national registry (OPCS) a further attrition – at least up to 3% – may arise (Swerdlow et al., 1993). We have previously shown (Johnson & Warnakulasuriya, 1991) that oral cancer in England and Wales (based on OPCS data) may be at least half as common as cervical cancer, a leading cancer afflicting the population. Based on the current findings it appears that the relative frequency of oral cancer in the UK could be much higher than reflected in the national estimates for this period. Indeed, if 'correction factors' of 16% for 1976–80 and 36% for 1981–87 are applied to the relatively static OPCS figures for oral cancers in England and Wales (Johnson & Warnakulasuriya, 1993) a rising incidence similar to rising mortality trends described for much of the rest of Europe (La Vecchia et al., 1992) is found. This present study shows that cancer registration was less than 100% efficient in the past. This represents a historical position, and audits such as the present one are necessary to monitor the improvements which are thought to be occurring. Methods of improving data collection of cancers treated by specialised units outside recognised radiotherapy departments and among patients managed as out-patients are being explored.

We thank Dr D. Pheby of the South Western Regional Cancer Registry, for providing access to his database and Mrs Anne Fisher, Supervisor, SWCR, for assistance. We thank Mrs Hilary Cavagnoli for secretarial support. K.A.A.S.W. is supported by the Dunhill Medical Trust.

### References

ADAMI, H., MALKER, B., HOLMBERG, L., PERSSON, I. & STONE, B. (1986). The relationship between survival and age at diagnosis in breast cancer. *N. Engl. J. Med.*, **315**, 559–563.

BENN, R.T., LECK, I. & NWENE, U.P. (1982). Estimation of completeness of cancer registration. *Int. J. Epidemiol.*, **11**, 362–367.

BOYLE, P., MACFARLANE, G.F., MAISONNEVE, P., ZHENG, T., SCULLY, C. & TEDESCO, B. (1990). Epidemiology of mouth cancer in 1989: A review. *J. R. Soc. Med.*, **83**, 724–730.

CANCER RESEARCH CAMPAIGN. (1993). *Oral Cancer*, Factsheet No. 14. CRC:London.

DARBY, S.C., O'HAGAN, J.A., KENDALL, G.M., DOLL, R., FELL, T.P. & MUIRHEAD, C.R. (1991). Completeness of follow up in a cohort study of mortality using the United Kingdom National Health Service Central Registers and records held by the Department of Social Security. *J. Epidemiol. Commun. Hlth*, **45**, 65–70.

HAWKINS, M.M. & SWERDLOW, A.J. (1992). Completeness of cancer and death follow-up obtained through the National Health Service Central Register for England and Wales. *Br. J. Cancer*, **66**, 408–413.

HMSO (1992). *The Health of the Nation: Strategy for Health in England*. HMSO: London.

HUNT, K. & COLEMAN, M.P. (1987). The completeness of cancer registration in follow-up studies – a cautionary note. *Br. J. Cancer*, **56**, 357–359.

JOHNSON, N.W. & WARNAKULASURIYA, K.A.A.S. (1991). Oral cancer: is it more common than cervical? *Br. Dent. J.*, **170**, 170–171.

JOHNSON, N.W. & WARNAKULASURIYA, K.A.A.S. (1993). Epidemiology and etiology of oral cancer in the United Kingdom. *Comm. Dent. Hlth*, **10** (Suppl 1), 13–29.

LA VECCHIA, C., LUCCHINI, F., NEGRI, E., BOYLE, P., MAISONNEUVE, P. & LEVI, F. (1992). Trends of cancer mortality in Europe, 1955–1989. *Eur. J. Cancer*, **28**, 132–235.

LANGDON, J.D. (1991). Oral cancer: is it more common than cervical? (letter). *Br. Dent. J.*, **170**, 287–288.

MACFARLANE, G.J., BOYLE, P. & SCULLY, C. (1992). Oral cancer in Scotland: changing incidence and mortality. *Br. Med. J.*, **305**, 1121–1123.

NWENE, U. & SMITH, A. (1982). Assessing completeness of cancer registration in the North-Western region of England by a method of independent comparison. *Br. J. Cancer*, **46**, 635–639.

SWERDLOW, A.J., DOUGLAS, A.J., VAUGHAN HUDSON, G. & VAUGHAN HUDSON, B. (1993). Completeness of cancer registration in England and Wales: an assessment based on 2,145 patients with Hodgkin's disease independently registered by the British National Lymphoma Investigation. *Br. J. Cancer*, **67**, 326–329.

THAMES CANCER REGISTRY (1992). *Cancer in South East Thames 1987–1989*. TCR:UK.

VILLARD-MACKINTOSH, L., COLEMAN, M.P. & VESSEY, M.P. (1988). The completeness of cancer registration in England: an assessment from the Oxford-FPA contraceptive study. *Br. J. Cancer*, **58**, 507–511.

WORLD HEALTH ORGANIZATION (1990). *International Classification of Diseases for Oncology*, 2nd edn. Percy, C., Van Holten, V. & Muir, C.C. (eds). WHO: Geneva.